# Multipotent adult progenitor cells decrease cold ischemic injury in *ex vivo* perfused human lungs: an initial pilot and feasibility study

**DOI:** 10.1186/2047-1440-3-19

**Published:** 2014-11-01

**Authors:** Saverio La Francesca, Anthony E Ting, Jason Sakamoto, Jessica Rhudy, Nicholas R Bonenfant, Zachary D Borg, Fernanda F Cruz, Meagan Goodwin, Nicholas A Lehman, Jennifer M Taggart, Robert Deans, Daniel J Weiss

**Affiliations:** Cardiac Surgery and Cardiopulmonary Transplantation, DeBakey Heart and Vascular Center, The Houston Methodist, Houston, TX USA; Athersys Inc, Cleveland, OH USA; Department of Nanomedicine, Houston Methodist Research Institute, Houston, TX USA; Department of Medicine, University of Vermont College of Medicine, 226 Health Science Research Facility, Burlington, VT USA; Federal University of Rio de Janeiro, Rio de Janeiro, Brazil; Harvard Apparatus Regenerative Technology, Inc, Holliston, MA USA

**Keywords:** Lung transplantation, Ischemia-reperfusion injury, Cell therapy, Mesenchymal stromal cell

## Abstract

**Background:**

Primary graft dysfunction (PGD) is a significant cause of early morbidity and mortality following lung transplantation. Improved organ preservation techniques will decrease ischemia-reperfusion injury (IRI) contributing to PGD. Adult bone marrow-derived adherent stem cells, including mesenchymal stromal (stem) cells (MSCs) and multipotent adult progenitor cells (MAPCs), have potent anti-inflammatory actions, and we thus postulated that intratracheal MAPC administration during donor lung processing would decrease IRI. The goal of the study was therefore to determine if intratracheal MAPC instillation would decrease lung injury and inflammation in an *ex vivo* human lung explant model of prolonged cold storage and subsequent reperfusion.

**Methods:**

Four donor lungs not utilized for transplant underwent 8 h of cold storage (4°C). Following rewarming for approximately 30 min, non-HLA-matched allogeneic MAPCs (1 × 10^7^ MAPCs/lung) were bronchoscopically instilled into the left lower lobe (LLL) and vehicle comparably instilled into the right lower lobe (RLL). The lungs were then perfused and mechanically ventilated for 4 h and subsequently assessed for histologic injury and for inflammatory markers in bronchoalveolar lavage fluid (BALF) and lung tissue.

**Results:**

All LLLs consistently demonstrated a significant decrease in histologic and BALF inflammation compared to vehicle-treated RLLs.

**Conclusions:**

These initial pilot studies suggest that use of non-HLA-matched allogeneic MAPCs during donor lung processing can decrease markers of cold ischemia-induced lung injury.

**Electronic supplementary material:**

The online version of this article (doi:10.1186/2047-1440-3-19) contains supplementary material, which is available to authorized users.

## Background

Primary graft dysfunction (PGD) affects an estimated 10% to 25% of lung transplant recipients and is the leading cause of early posttransplantation morbidity and mortality [1]. PGD, defined by poor oxygenation in the immediate and early postoperative period, up to 72 h after transplantation, results from multiple pathologic mechanisms including donor lung ischemia, cold static organ preservation, and lung ischemia-reperfusion injury (IRI) [2]. The mechanisms of lung IRI have been extensively investigated, and a common pathway is activation of inflammatory and immune mediators that are damaging to the lung allograft [3, 4]. Thirty-day mortality rates are up to eightfold higher in lung transplant patients with severe PGD as compared with those without PGD. Associated signs and symptoms include interstitial/alveolar edema, increased pulmonary vascular resistance, intrapulmonary shunting, decreased lung compliance, and diffuse alveolar damage (DAD). Both PGD and DAD are associated with an earlier onset of bronchiolitis obliterans syndrome (BOS) and chronic rejection [5–12]. Despite recent advances in donor lung preservation [13], new therapeutic approaches to reduce IRI and thus both PGD and chronic rejection are desperately needed [14].

Adherent stromal (stem) cells isolated from bone marrow, adipose, and other sources, including mesenchymal stromal (stem) cells (MSCs) and multipotent adult progenitor cells (MAPCs), have potent immunomodulatory properties and have been increasingly investigated in a range of inflammatory and autoimmune conditions [15–17]. Notably, both systemic and intratracheal MSC administration reduces inflammation and injury in a wide spectrum of preclinical lung injury models [18, 19]. Further, MSCs and MAPCs have limited immunogenicity, and thus, use of non-HLA-matched MAPCs and MSCs is increasingly found to be safe and potentially efficacious in a growing spectrum of clinical disease investigations including lung diseases [15–17]. Postulated mechanisms include release of anti-inflammatory cytokines and conversion of macrophages to anti-inflammatory M2 phenotype [15–17, 20]. These properties make MSCs and MAPCs particularly interesting for use as a cellular therapy in solid-organ transplantation [21, 22].

We hypothesized that administering MSCs or MAPCs to donor lungs would decrease potential lung inflammation resulting from storage and handling prior to implantation and thus decrease IRI with subsequent decrease in both PGD and in chronic rejection. To initially assess this in a preclinical translational pilot and feasibility study, we determined the impact of intratracheal administration of non-HLA-matched human bone marrow-derived allogeneic MAPCs, a cell type currently being investigated in clinical trials in other diseases [23–27] (MultiStem®, Athersys Inc., Cleveland) in four human donor lungs not suitable for transplantation using an *ex vivo* human lung model of cold ischemia and subsequent reperfusion that reproduces current approaches to organ procurement and storage.

## Methods

### Lung harvest and *ex vivo*perfusion

**Figure 1:**
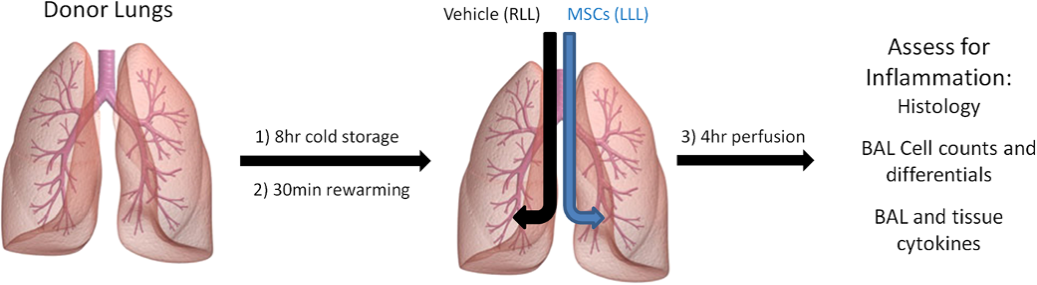
**Schematic of study design.**

Following informed consent, donor lungs not suitable for clinical use were procured under an established IRB protocol at The Houston Methodist (Houston Methodist Institutional Review Board protocol (2)1111-0205). The lungs were perfused with Perfadex (Vitrolife AB, Gothenburg, Sweden) and then stored in a refrigerator at 4°C for a total of 8 h. *Ex vivo* lung perfusion (EVLP) was then performed with a CE-marked Vivoline LS1 perfusion system (Vivoline Medical AB, Lund, Sweden) utilizing pH-adjusted Steen Solution (Vitrolife AB) containing meropenem 100 mg (AstraZeneca AB, Sodertalje, Sweden) and 10,000 U of heparin (LEO Pharmaceutical, Copenhagen, Denmark) (Figure 1). The lungs were initially perfused at 0.5 L/min and then warmed over 30 min to a target of 36°C with the flow rate increased gradually to a target of 70 mL/min per kilogram of donor weight. When the perfusate temperature reached 32°C, volume-controlled mechanical ventilation (Hamilton C2, Bonaduz, Switzerland) was started at an initial tidal volume of 3 mL per kilogram of donor weight with a positive end-expiratory pressure (PEEP) level of 5 cm H_2_O, a rate of 7–10 breaths/min, and a FiO_2_ of 0.5. Tidal volume was then increased gradually to a maximum of 7 mL per kilogram of donor weight.

### Cells and cell inoculations

Human bone marrow-derived MAPCs were isolated from a single bone marrow aspirate, obtained with consent from a healthy donor, and processed and extensively characterized according to previously described methods [23–27]. In brief, MAPCs were cultured in fibronectin-coated plastic tissue culture flasks under low oxygen tension in a humidified atmosphere of 5% CO_2_. Cells were cultured to subconfluence in MAPC culture media (low-glucose DMEM [Life Technologies Invitrogen] supplemented with FBS [Atlas Biologicals, Fort Collins, CO], ITS liquid media supplement [Sigma], MCDB [Sigma], platelet-derived growth factor [R&D Systems, Minneapolis, MN], epidermal growth factor [R&D Systems], dexamethasone [Sigma], penicillin/streptomycin [Life Technologies Invitrogen], 2-phospho-L-ascorbic acid [Sigma, St. Louis, MO], and linoleic acid-albumin [Sigma]). Cells were passaged every 3–4 days, harvested using trypsin/EDTA (Life Technologies Invitrogen, Carlsbad, CA). The cells were positive for CD49c (90%) and CD90 (90%) and negative for MHC class II (<1%) and CD45 (<1%) (all Abs were from BD Biosciences, Franklin Lakes, NJ). Cells were subsequently cryopreserved at population doubling 30–35 in cryovials in the vapor phase of liquid nitrogen at a concentration of 1–10 × 10^6^ in 1 mL (PlasmaLyte, 5% HSA and 10% DMSO). Immediately prior to their use, MAPCs were thawed and used directly. As per previous testing, there is no apparent loss of function of the MAPCs immediately following thawing in appropriate *in vitro* assays. All the studies performed to date with MAPC have used cells that have been cryopreserved and used directly after thawing. These include 16 publications as well as the utility in four clinical trials for AMI, GvHD, acute stroke, and ulcerative colitis [23–28].

Dose was determined based on prior studies with a sheep model for ARDS that also utilized intrabronchial delivery of MAPC [29]. Each study lung received a dose of 10^7^ MAPCs. Another lung inadvertently received a lower dose (10^6^ MAPCs) and so was not included in the overall assessments. Given the relatively small number of lungs in this pilot, we utilized an approach in which each lung was its own control, comparing cell administration in one lobe to vehicle administration in the contralateral lobe. When each lung temperature reached approximately 32°C, cells were thawed, diluted into 19 mL of sterile saline, and administered by a bronchoscope into the proximal portion of the left lower lobe (LLL) bronchus. A similar volume of vehicle (20 mL of sterile saline) was similarly inoculated into the proximal portion of the right lower lobe (RLL) bronchus. Mechanical ventilation was initiated 5 min after cell or vehicle delivery.

### BAL fluid and tissue analyses

Tissue biopsies were taken using a mechanical stapler (Covidien DST Series™ GIA™ Staplers) prior to and 2 or 4 h after cell or vehicle infusions. Care was taken to obtain biopsies from the same areas, corresponding to the areas of cell or vehicle instillations. After 4 h of perfusion, the inoculated regions of the RLL and LLL were lavaged with 60 mL of saline and the BAL fluid assessed for total cell counts and cell differentials by three blinded observers, protein content, and levels of inflammatory cytokines determined by cytokine antibody array and ELISAs (R&D Systems, Minneapolis, MN) [30, 31]. The lungs were then fixed in 10% formalin for 1 h and a minimum of three to four biopsies taken from the areas of instillation. Mounted 5-μm paraffin sections of each biopsy piece were evaluated for inflammation on ten airways per animal in a blinded fashion by three individuals using an established semi-quantitative scoring system as previously described [30, 31]. Lung biopsy samples were homogenized and assessed for content of inflammatory cytokine mRNA by qPCR.

### Statistical analyses

Groups were compared using either one-way or two-way ANOVA with a Fisher’s LSD posttest or by direct analysis between two groups by Student’s *T*-test, using a Welch’s correction for unequal variances, as appropriate [30, 31].

Full details for all methods are in Additional file 1.

## Results

The relevant clinical characteristics of the donor lungs are summarized in Table 1. Donor age ranged from 44 to 66, and two of the four donor lungs were obtained from patients with devastating neurologic events, one from asphyxia, and one from a motor vehicle accident. All of the lungs were not deemed suitable for transplant because of poor functional status including low PaO_2_ values with a mean of 184.8 ± 49.3 mmHg at 100% FiO_2_ at a PEEP of 10 mmHg. These lungs also had radiographic abnormalities, variously including contusions, significant emphysema, or lobar collapse that did not respond to recruitment maneuvers in the operating room. All of the lungs also had radiographic signs of pulmonary edema with two having also pleural effusion, and all were noted to be variably edematous following surgical removal. Lung #4 had RLL collapse on CXR but expanded following removal and bronchoscopic removal of mucus plugs.

**Table 1:** **Clinical characteristics of the donor lungs**

Donor characteristics	1	2	3	4	Mean ± SD
Age	55	56	44	50	51.25 ± 5.5
Sex	Male	Male	Male	Male	
Cause of death	CVA	SH	Asphyxiation	MVA	
PaO_2_ at 100% FiO_2_	150	186	254	149	184.8 ± 49.3
PEEP	10	10	10	10	10 ± 0
Radiographic findings	Infiltrate-edema	Infiltrate-edema	Infiltrate-edema	Edema-right lower lobe collapse, right pleural effusion	
Lung appearance	Edematous	Edematous	Edematous	Contusions, edematous	

*CVA* cerebrovascular accident, *SH* subarachnoid hemorrhage, *MVA* motor vehicle accident.

**Figure 2:**
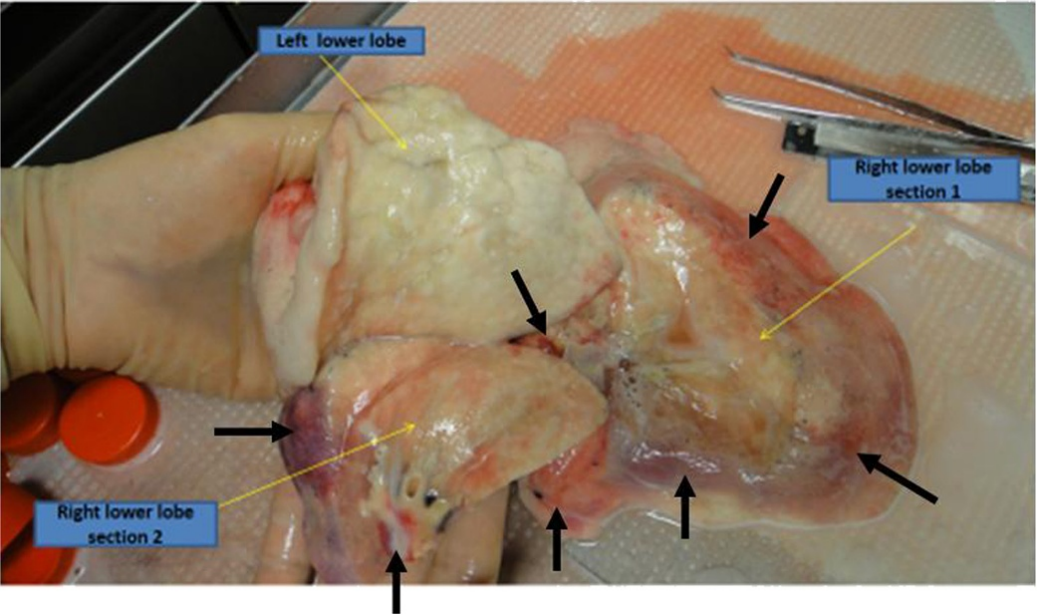
**Representative gross appearances of the RLL and LLL of lung 2 following reperfusion.** The MAPC-treated LLL appears normal while the vehicle-treated RLL appears edematous and inflamed. Black arrows indicate areas of edema and inflammation in the RLL.

A summary of the protocol utilized for each lung is presented in Table 2 and also in schematic form in Figure 1. Given the small number of lungs utilized for this pilot study, we chose to directly compare right to left lower lobes in each lung. Appreciating that any potential lung damage might be heterogenous, as could be best determined by gross appearance and radiographic assessments, the right vs left lower lobes were fairly similar for each of the lungs. Overall, the lungs had similar cold storage (8 h) and rewarming (24.8 + 2.5 min) times and subsequently similar reperfusion times (3.6 ± 0.8 h) following bronchoscopic administration of cells or vehicle. At the end of the reperfusion period, there was some degree of further edema that had developed in each lung. However, overall, there was less visible edema and inflammation in the MAPC-treated (LLL) vs vehicle-treated (RLL) lobes. Representative images are shown in Figure 2.

**Figure 3:**
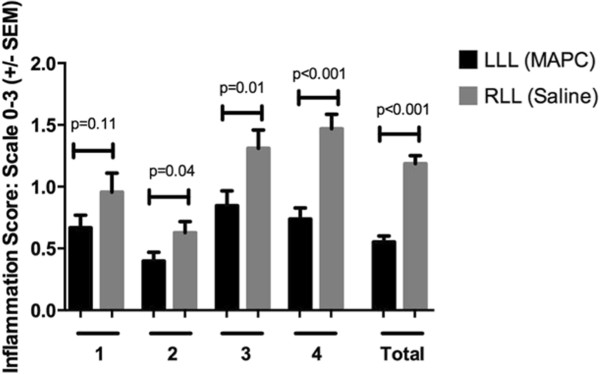
**Semi-quantitative scoring demonstrates significant decrease in overall inflammation in the MAPC-treated LLL compared to the vehicle-treated RLL in three out of four lungs and in aggregate.** Means ± SD of pooled observations from three blinded observers are depicted.

**Figure 4:**
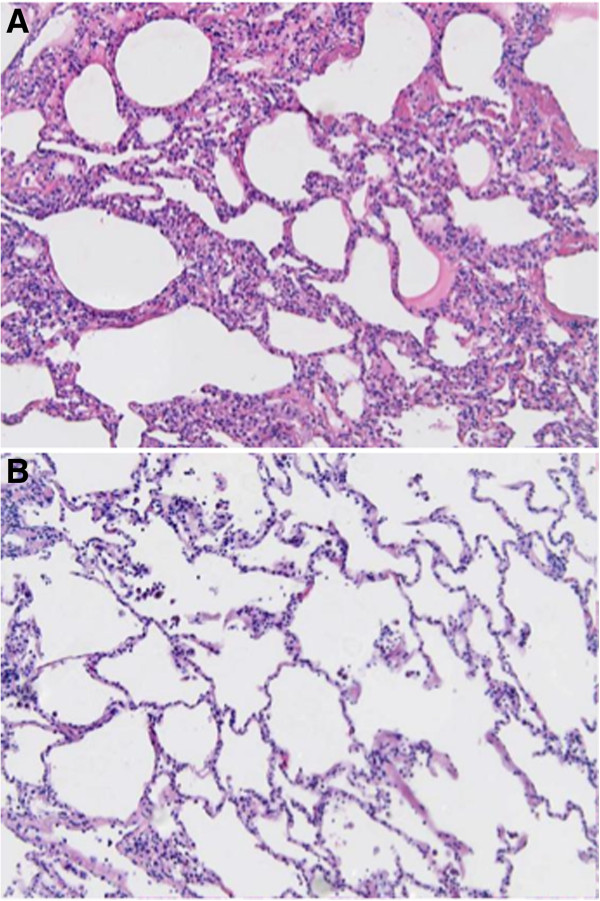
**Representative photomicrographs from lung 1 demonstrate (A) alveolar septal thickening, edema, and perivascular and peri-bronchial inflammatory cell infiltrates in the control-treated RLL vs (B) minimal to no significant inflammation in MAPC-treated LLL.** Original Mag 200 × .

Histologic assessment of the lungs at the end of the reperfusion period demonstrated that although patches of inflamed areas could be found in some of the MAPC-treated LLLs, there was significantly less overall inflammation in three out of the four lungs and also averaged over all four lungs, as assessed by semi-quantitative scoring of peribronchial, perivascular, and alveolar septal edema and by presence of inflammatory cell infiltrates (Figure 3). Representative photomicrographs are depicted in Figure 4.

**Figure 5:**
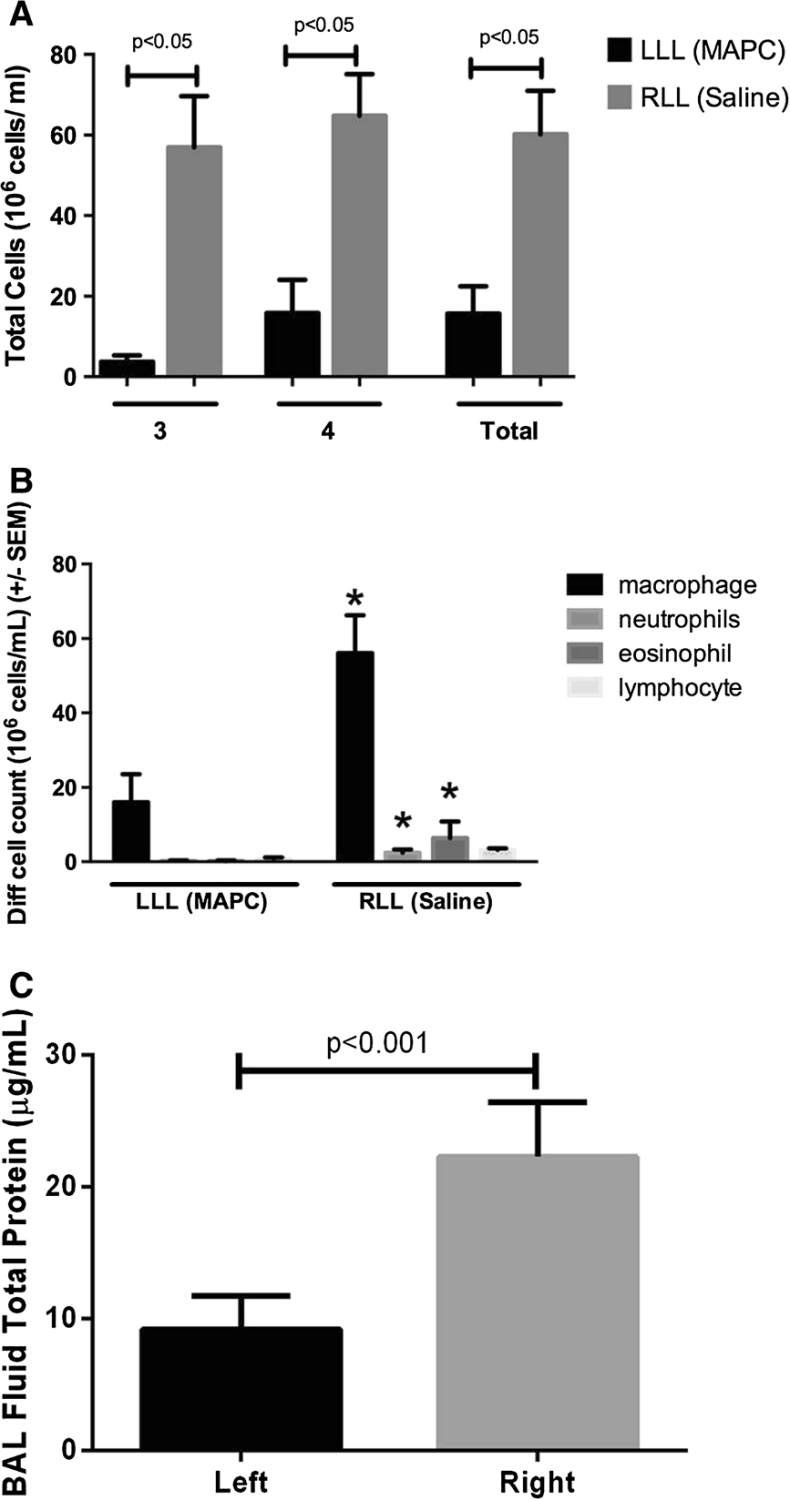
**BAL fluid analyses. (A)** Decrease in total BAL fluid cell counts in the MAPC-treated LLL in lungs 3 and 4. Total cell counts were not assessed in lungs 1 or 2. Data represents total cell counts for each individual lung and means ± SEM of the values obtained for each lung. **(B)** MAPC instillation also resulted in a significant decrease in the elevated numbers of BAL fluid total neutrophils and eosinophils in all four lungs. Data represents means ± SEM of pooled observations from three blinded observers. *Significantly different from the corresponding LLL value. **(C)** BAL fluid total protein levels were lower at the end of the perfusion period in the MAPC-treated LLL vs vehicle-treated RLL. Data represents means ± SEM.

Total BAL fluid cell counts were obtained in two out of four lungs (lungs 3 and 4). In both cases, there was a decrease of total BAL fluid cells in the MAPC-treated LLL compared to the vehicle-treated RLL (Figure 5A). Total BAL fluid cell counts were not obtained in the other lungs (lungs 1 and 2) due to inadvertent laboratory error. Cell differentials obtained on BAL fluid samples from all four lungs demonstrated a consistent increase in neutrophils and eosinophils in the vehicle-treated RLL that was ameliorated in the MAPC-treated LLL (Figure 5B). Measurements of BAL fluid total protein levels was variable between the lungs, but a consistent decrease in total protein in the MAPC-treated LLL vs vehicle-treated RLL was observed in all four lungs (Figure 5C).

**Figure 6:**
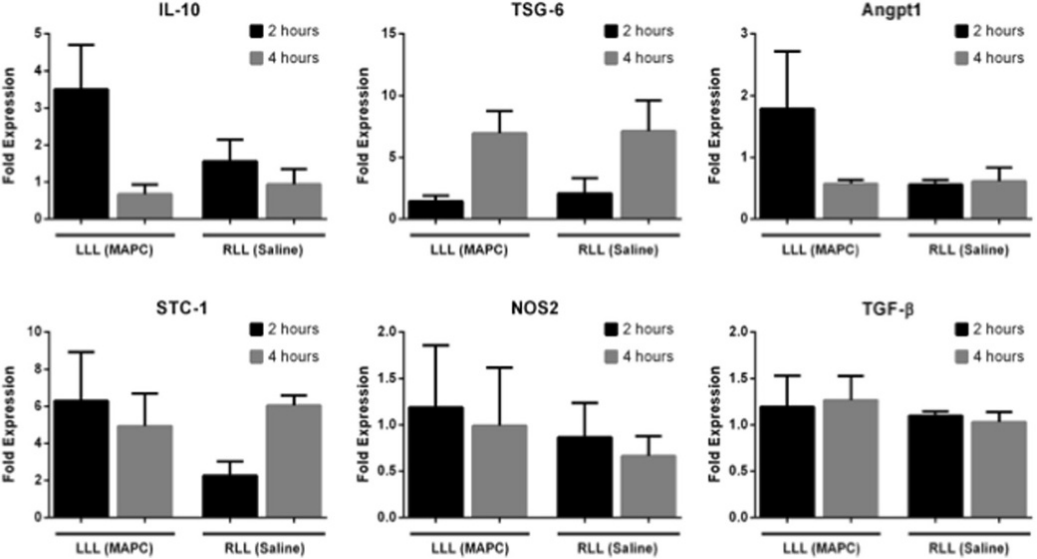
**Cytokine analysis of lung tissue.** qPCR analysis was performed on the lung tissue samples collected from the MAPC-treated LLL and vehicle-treated RLL of lungs 2–4 at *t* = 0, 2, and/or 4 h. The fold expression represents the levels of the target gene compared to the *t* = 0 value. All data were normalized to a housekeeping gene, GAPDH. Data represents means ± standard deviations from LLL and RLL samples from lungs 2–4. Although strong trends towards differences at 2 h were observed in several of the measured mRNA levels of mediators typically associated with MSC immunomodulation, none reached statistical significance.

Levels of BAL fluid cytokines and chemokines demonstrated substantial variability between the different lungs. This included soluble anti-inflammatory mediators implicated in preclinical models of MAPC actions in lung injury and other models, such as IL-1RA, IL-10, STC, TGS-6, and iNOS. None of these were reliably or consistently increased in the MAPC-treated LLL in any of the four lungs (data not shown). Tissue mRNA levels were assessed in three of four lungs (lungs 2–4) by qPCR analyses of biopsy samples obtained prior to cell or vehicle administration and then after either 2 or 4 h of reperfusion period. Overall, patterns of tissue mRNA levels were more consistent between the three lungs. Comparable to BAL fluid levels of IL-10 protein, there was a strong early trend in elevated IL-10 in the LLL with a 3.5-fold increase in the levels of tissue IL-10 mRNA in the MAPC-treated LLL compared to only a 1.6-fold increase in vehicle-treated RLL as assessed at 2 h (Figure 6). Similar trends towards increases in LLL vs RLL were also observed at 2 h in mRNA levels of Angpt1 and STC1. Interestingly, for both the LLL and RLL, there was a large increase in the fold expression of TSG6 from 2 to 4 h. No clear patterns of changes were observed in mRNA levels of the other chemokines and cytokines evaluated including TGFβ and NOS2.

**Table 2:** **Summary of experimental protocol**

Donor lung	1	2	3	4	Mean ± SD
Duration of cold static storage (h)	8	8	8	8	8.0 ± 0
Rewarming time (min)	22	25	28	24	24.8 ± 2.5
Duration of *ex vivo* perfusion (h)	4	2.5	4	4	3.6 ± 0.8
Cells or vehicle delivered	10^7^ MSC to LLL	10^7^ MSC to LLL	10^7^ MSC to LLL	10^7^ MSC to LLL	
	Vehicle to RLL	Vehicle to RLL	Vehicle to RLL	Vehicle to RLL

## Discussion

In an initial small-scale pilot and feasibility study, we found that airway administration of bone marrow-derived MAPCs decreased a number of inflammatory markers provoked by prolonged cold storage and subsequent reperfusion in four suboptimal donor lungs. These results suggest that further studies to more fully investigate the potential anti-inflammatory effects of MAPCs and MSCs in models of IRI are warranted.

A number of different methods have been studied to improve the viability of donor lungs and to decrease either warm or cold ischemic inflammatory injury. These include a flushing solution with extracellular characteristics delivered both in an antegrade and in a retrograde fashion and the use of a portable *ex vivo* preservation system currently under clinical investigation for use during transport of donor lungs [14]. Different areas of research for therapeutic interventions aim to modulate the response induced by ischemia and reperfusion. For example, experimental animal models have shown a beneficial effect from gene therapy delivery of IL-10 [32] and from adenosine receptor activation [33, 34]. However, while the experimental data are promising, it is unlikely that modulating one out of many inflammatory pathways can regulate a phenomenon that alters several cellular mechanisms involved, as innate and adaptive immunity, the activation of the complement cascade, endothelial dysfunction, and the triggering of cell death. In contrast, bone marrow-derived MSCs and MAPCs have the unique potential of acting on multiple inflammatory pathways involved in ischemia/reperfusion injury.

Systemic or intratracheal administration of bone marrow-derived MSCs or MAPCs results in decreased inflammation and deleterious immune responses in a wide range of preclinical inflammatory disease models including experimentally induced acute lung injuries in mouse and in *ex vivo* perfused human lung models [18, 19]. Furthermore, accumulating data in a growing number of clinical trials suggest that systemic administration of non-HLA-matched allogeneic MSCs and MAPCs is safe [23–27, 35–37]. Thus, MSCs or MAPCs might conceivably be utilized to decrease IRI in donor human lungs with subsequent decrease in incidence of primary graft dysfunction and longer term complications of lung transplantation.

EVLP was originally designed as a method to assess the quality of lungs from donation after cardiac death (DCD) and from other nonacceptable donor lungs [38, 39]. This technique is currently under clinical trial using asanguinous perfusates for the evaluation and reconditioning of potential donor lungs that under current criteria are not deemed suitable for transplant [40]. EVLP with asanguinous perfusates thus further offers an opportunity to administer MSCs or MAPCs directly into the donor lung by either intratracheal or intravascular routes prior to implantation. In an initial proof-of-concept pilot and feasibility study using an explant human lung model of cold ischemia and subsequent rewarming and reperfusion, a simple approach involving bronchoscopic instillation of bone marrow-derived MAPCs significantly decreased markers of inflammation and injury. Intriguingly, a similar reduction in inflammatory endpoints was also observed in the lower lobe of an additional lung in which tenfold fewer MAPCs were administered to the LLL (data not shown). These results suggest that MAPC or MSC administration to donor lungs may be a useful approach to both decrease PGD and also improve viability of suboptimal donor lungs. Indeed, a recent parallel study demonstrated that MSC administration reduced alveolar edema in failed donor human lungs [41]. We chose to initially assess direct airway MAPC administration into a single lobe with the contralateral lung as comparison to directly assess effects within each individual lung. This was done as the initial injuries to these areas appeared comparable and this was a straightforward approach to use for a pilot study. In particular, lower lobes tend to be most often affected by aspiration and other damage in ventilated brain-dead potential donors. Overall, the major advantage of this approach was the ability to obtain useful pilot data out of a smaller number of lungs. However, as the nature and degree of lung injury can be heterogenous, future larger scale studies will use complementary approaches such as MAPC vs vehicle administration in alternating whole lungs. With larger scale studies, the lungs can be grouped accordingly to a number of clinical factors including radiographic infiltrates, P/F ratio, cause of death, and length of ICU stay for example.

The cold ischemic storage (8 h of total cold storage) is comparable to currently utilized clinical approaches. We also chose to use “off the shelf” non-HLA-matched MAPCs as proof of feasibility. Given the short time period in which transplantation needs to occur after identification of a donor lung and suitable recipient, it is not yet feasible to consider isolating and expanding MSCs or MAPCs from either the donor or recipient. Further, it is conceivable that MSCs or MAPCs obtained from patients with lung diseases may be adversely affected by the underlying pathophysiologic mechanisms. Nonetheless, given clinical variability between lungs and lobes within each lung and the relatively small sample size, the current pilot data is still compelling in its consistency of an ameliorating effect of MAPC administration in reducing BAL fluid total cell counts, protein, and histologic inflammation.

These findings need to be further explored, clarified, and validated in a larger number of lungs and under different paradigms of cold storage injury conditions, dose and timing of cell administration, and experimental approaches involving MAPC vs vehicle administration to alternating lungs as compared to lobar comparisons within each lung. Future studies will also investigate conditioned media and microsomal particles produced by the cells to see if these can convey similar effects. Physiologic endpoints including alveolar fluid clearance, as recently demonstrated to be improved by MSC administration in *ex vivo* perfused human lungs [40], and oxygenation will be included. Future studies will further assess MSC effects on endothelial injury in the perfused lungs. We did not assess localization, clearance or removal of the MAPCs from the donor lungs, or the effect of the lung environment on MAPC function, but these also need to be evaluated. Assessment in preclinical models of lung transplantation will also provide critical correlative information. If the pilot results are validated, this approach may be a simple and effective means of decreasing IRI.

## Electronic supplementary material

Additional file 1: **Supplemental materials and methods.** (DOCX 20 KB)
