# Supplementary material for: Multipotent adult progenitor cells decrease cold ischemic injury in ex vivo perfused human lungs: an initial pilot and feasibility study
Source: Transplant Res. 2014 Nov 1;3:19. doi: 10.1186/2047-1440-3-19 (PMC4323223; doi:10.1186/2047-1440-3-19)
Supplement: Supplementary file 1 — Additional file 1: Supplemental materials and methods. (DOCX 20 KB) [file 13737_2014_60_MOESM1_ESM.docx]

**Supplemental Materials and Methods**

*Lung harvest and ex vivo perfusion*

Following research consent obtained by the local Organ Procurement Agency, LifeGift, the discarded donated lungs were procured for this study under an established IRB protocol at the Houston Methodist (IRB(2)1111-0205). Lungs from each of the five patients were procured in a standard fashion with antegrade Perfadex (Vitrolife AB, Gothenburg, Sweden) 60 ml/Kg flush plus retrograde Perfadex perfusion through the pulmonary veins. The lungs were then stored in plastic bags containing 1 liter of Perfadex and were kept on ice during transport. Once the lungs arrived at the Houston Methodist, they were then stored in a refrigerator @ 4ºC for a total 8 hours of cold static storage in order to induce cold ischemic injury.

*Ex Vivo* Lung Perfusion (EVLP) was performed with the CE-marked Vivoline LS1 (Vivoline Medical AB, Lund, Sweden) (**Figure 1**) (35-37). The system was primed with 2.5 L of Steen Solution (XVIVO Perfusion). The use of washed red blood cell or blood was avoided in order to decrease the number of variables in the feasibility study. Meropenem 100 mg (AstraZeneca AB, Sodertalje, Sweden) and 10,000 U of Heparin (LEO Pharmaceutical, Copenhagen, Denmark) were added to the perfusate. Before the lungs were connected to the EVLP unit, the pH in the solution was corrected to between 7.35 and 7.45 using trometamol (Addex-THAM, Fresenius Kabi AB, Uppsala, Sweden). In one case where the heart was procured as well, a Dacron Graft was sutured to the divided pulmonary artery branches in order to reconstitute the integrity of the pulmonary artery (PA) and facilitate the connection of the lung to the EVLP circuit. The trachea was connected to the mechanical ventilator via a silicon tube size matching the tracheal diameter. A temperature probe was positioned inside the left atrium. Initially, for de-airing the circuit, the lungs were perfused at a flow rate of 0.5 L/min. The shunt for de-airing on the inflow cannula was kept open until the organ reached 32 degrees and then closed for the rest of the session. The flow was then increased to 100% of estimated cardiac output for the specific set of lungs. The lungs were then warmed over 30 minutes to a target of 36ºC and the temperature difference between lung blood inflow and outflow was not allowed to exceed 8ºC. The flow rate was then increased gradually to a target level of 70 mL/min per kilogram donor weight, during which the PA pressure was measured continuously and limited to 15 mm Hg. Rewarming was achieved within 20-30 minutes. When the perfusate temperature reached 32ºC, mechanical ventilation was started in volume-controlled mode at an initial tidal-volume of 3 ml per kilogram of donor weight with a positive end-expiratory pressure (PEEP) level of 5 cm H2O, a rate of 7-10 breaths/min, and a FiO_2_ of 0.5. Tidal volume was then increased gradually to a maximum of 7 mL per kilogram of donor weight. Perfusate samples for blood gas analyses were drawn from the dedicated port of the system.

*Cells*

Human bone marrow derived MAPCs were isolated from a single bone marrow aspirate, obtained with consent from a healthy donor, and processed according to previously described methods (23-25). In brief, MAPCs were cultured in fibronectin-coated plastic tissue culture flasks under low oxygen tension in a humidified atmosphere of 5% CO_2_. Cells were cultured in MAPC culture media (low-glucose DMEM [Life Technologies Invitrogen] supplemented with FBS (Atlas Biologicals, Fort Collins, CO), ITS liquid media supplement [Sigma], MCDB [Sigma], platelet-derived growth factor (R&D Systems, Minneapolis, MN), epidermal growth factor (R&D Systems), dexamethasone (Sigma], penicillin/ streptomycin [Life Technologies Invitrogen], 2-Phospho-L-ascorbic acid [Sigma, St. Louis, MO), and linoleic acid-albumin (Sigma). Cells were passaged every 3-4 d, harvested using trypsin/EDTA (Life Technologies Invitrogen, Carlsbad, CA). The cells were positive for CD49c and CD90 and negative for MHC class II and CD45 (all Abs were from BD Biosciences, Franklin Lakes, NJ). Cells were subsequently frozen at population doubling 30-35 in cryovials in the vapor phase of liquid nitrogen at a concentration of 1-10 x 10^6^ in 1 ml (PlasmaLyte, 5% HSA and 10% DMSO). Immediately prior to their use, MAPCs were thawed and used directly.

*Cell inoculations, lung incubations, and BAL fluid analyses*

When the temperature as measured by the intra-atrial probe reached approximately 32ºC, MultiStem 1 ml vials were thawed, diluted into 19 ml of sterile saline and administered by bronchoscope into the proximal portion of the LLL bronchus. A similar volume of vehicle (20 ml of sterile saline) was similarly inoculated into the proximal portion of the RLL bronchus. Five minutes after delivery of MultiStem, the lungs were connected to a Hamilton-C2 mechanical ventilator. After either 2 or 4 hours of perfusion on the Vivoline system the experiments were stopped. Five minutes before stopping the perfusion, the same subsegments of the RLL and LLL that had been previously inoculated with either cells or vehicle were lavaged with 60 mL saline. The recovered BAL fluid was then separated into aliquots of either raw BAL fluid for assessing total cell counts and cell differentials or was centrifuged (1200g x 10 min at 4ºC) and the supernatant was collected in separate tubes, snap frozen, and stored at -70ºC (27,28). For one lung, BAL fluid samples were also obtained during rewarming phase before ventilation was started, just prior to MSC or vehicle delivery

Total BAL fluid cell numbers were determined using an ADVIA® Hematology Analyzer (Siemens Diagnostics, Johnson City, TN). Cytospins were made using 5x10^4^ cells centrifuged onto pre-cleaned, pre-treated glass slides (Corning Incorporate, Corning, NY) at 800 rpm for 8 min, dried overnight, and stained using DiffQuick (Hema 3 Stain Set, Fisher Scientific, Pittsburgh, PA). Cell populations were determined by blinded manual count of 200 cells performed by three separate individuals (27,28). Protein content in undiluted BAL fluid was assessed by Bradford assay (Bio-Rad, Hercules, CA). The Human Cytokine Array Kit, Panel A (R&D Systems, Minneapolis, MN) was used to examine BAL fluid supernatants for soluble cytokines, chemokines, and other substances including C5/Ca, CD40L, CD54, CXCL1, CXCL10, G-CSF, Gro-1α, IL-1α, IL-1β, IL-1RA, IL-6, IL-8, IL-10, IL-16, IL-23, IP-10, I-TAC, MCP-1, MIF, PAI-1, RANES, serpin E1, sICAM, sTREM-1, TNFα, and the relative amount of cytokine compared to internal controls determined on a UVP Bioimaging system.(Upland, CA). Elisas for other specific cytokines were performed according to manufacturer’s instructions, IL-10 (R&D Systems, Minneapolis, MN, Cat#:D1000B), and STC1, TSG-6 and iNOS (MyBioSource, San Diego, CA, Cat#s: MBS946255, MBS926793, MBS723617).

*Histologic assessments*

Following BAL at the end of the perfusion period, the lungs were subsequently gravity fixed with 10% formalin at room temperature for 1 hour. Fixed lungs were dissected and the areas where cells were instilled stored in 10% formalin prior to paraffin fixation. Mounted 5 µm sections were then evaluated for histologic appearance. Lung inflammation was scored on 10 airways per animal, in a blinded fashion by three individuals, based on the presence and intensity of peri-bronchial cell infiltrates compared to known positive and negative controls using an established semi-quantitative scoring system, using a 0-3 range and 0.5 scale increments as previously described (27,28).

*qPCR analyses of tissue inflammatory markers*

Lung biopsy samples from lungs 2-5 were obtained using an automatic stapler (Covidien GIA™ DST Series™ 80mm) from the periphery of the LLL and RLL just prior to cell or vehicle infusion at 2 and 4 hours after cell or vehicle infusion and at the end of the experiment. The samples were snap frozen and subsequently homogenized and the expression levels of inflammatory cytokine mRNAs determined by qPCR (see details below).

Samples were homogenized in RNA lysis buffer and RNA extracted using the RNeasy kit (Qiagen, Germantown, MD) according to manufacturer’s instructions. Additional DNase treatment was performed using the DNA-free kit (Life Technologies, Carlsbad, CA). RNA concentration was measured by NanoDrop 2000 (Thermo Scientific, Waltham, MA) and 1 μg RNA was reverse transcribed using M-MLV Reverse Transcriptase (Promega, Madison, WI) followed by RNAse treatment using RNace-it Cocktail (Agilent, Santa Clara, CA). Reverse transcriptase negative samples and water were run as controls. 5 μl of the cDNA was mixed with SYBR green (Promega) and primers (IDT) and run on the ABI 7500 FAST system (Applied Biosystems, Foster City, CA). The samples were normalized to GAPDH and expressed as a percent of Human Reference (Agilent) +/- standard deviation.

Primer Sequences are as follows:

VEGFA –F 1- 5’ TGG TGT CTT CAG TGG ATG TAT TT 3’

VEGFA – R1 - 5’ AGT CTC TCA TCT CCT CCT CCT C 3’

IGF 1 –F4- 5’ GAA TCC TTC CTC TCC TTG GAA C 3’

IGF1 – R4 5’ GCC TTC TCC CAA GTG CAT AA 3’

EGF –F1- 5’ ACA CAT GCT AGT GGC TGA AA 3’

EGF-R1- 5’ GCA TCC TCT CCC TCT GAA ATA C 3’

IL-10 – F2- 5’ GCT GGA GGA CTT TAA GGG TTA C 3’

IL-10-R2- 5’ GAT GTC TGG GTC TTG GTT CTC 3’

FGF2-F1- 5’ GCT GGT GAT GGG AGT TGT ATT T 3’

FGF2-R1- 5’ CTG CCG CCT AAA GCC ATA TT 3’

HGF-F1- 5’ TGG GAA CCA GAT GCA AGT AAG

HGF-R1- 5’ AAT GAG TGG ATT TCC CGT GTA G 3’

CCL5-F1- 5’ TGC CCA CAT CAA GGA GTA TTT 3’

CCL5-R1- 5’ GAT GTA CTC CCG AAC CCA TTT 3’

TGFB1-F1- 5’ CGT GGA GCT GTA CCA GAA ATA C 3’

TGFB1-R1- 5’ CAC AAC TCC GGT GAC ATC AA 3’

CXCL10-F1- 5’ GTA ATA ACT CTA CCC TGG CAC TAT AA 3’

CXCL10-R1- 5’ CAT GGG AAA GGT GAG GGA AAT A 3’

NOS3-F2- 5’ CCG GAA CAG CAC AAG AGT TA 3’

NOS3-R2- 5’ GTC TGT GTT ACT GGA CTC CTT C 3’

STC1-F1- 5’ GGT CAA TGT CAA GAG AGG AAG AG 3’

STC1-R1- 5’ CTA GTG AGA GTC AAG CAC CAA TAG 3’

GAPDH-F1- 5’ GGT GTG AAC CAT GAG AAG TAT GA 3’

GAPDH-R1- 5’ GAG TCC TTC CAC GAT ACC AAA G 3’

ANGPT1-F2- 5’ CCA AAG AGG CCTG GAA GGA ATA 3’

ANGPT1-R2- 5’ GTA CTG CCT CTG ACT GGT AAT G 3’

NOS2-F1- 5’ GTC AGA GTC ACC ATC CTC TTT G 3’

NOS2-R1- 5’ GCA AGC TCA TCT CCA CAG TAT C 3’

TNFAIP6-F1- 5’ CAG GTT GCT TGG CTG ATT ATG 3’

TNFAIP6-R1- 5’ GCA AGC TCA TCT CCA CAG TAT C 3’

FGF7-F1- 5’ CTT GAG GTC AGC CTA CAG ATA AC 3’

FGF7-R1- 5’ ACC TCC CAT TGG TGA ACA TAT AA 3’
